# Antagonism of cytokine-induced eosinophil accumulation in asthma

**DOI:** 10.3389/fphar.2012.00197

**Published:** 2012-11-22

**Authors:** Garry M. Walsh

**Affiliations:** School of Medicine and Dentistry, Institute of Medical Sciences, University of AberdeenAberdeen, UK

Asthma is a chronic inflammatory condition of the airways characterized by reversible airway obstruction, airway hyperresponsiveness (AHR) to normally harmless stimuli and airway inflammation. Eosinophilic asthma is a phenotype of the condition characterized by increased blood or sputum eosinophils whose numbers correlate with disease severity. Release of their potent pro-inflammatory arsenal by infiltrating tissue eosinophils, including granule-derived basic proteins, mediators, cytokines and chemokines, contributes to airway inflammation, and lung tissue remodeling that includes airway thickening and fibrosis. More recent evidence suggests that in addition to their role as degranulating effector cells, eosinophils have the capacity to act as antigen presenting cells resulting in T cell proliferation and activation (Blanchard and Rothenberg, 2009). Eosinophil extravasation from the post-capillary venules, migration within the interstitium, cellular activation and tissue retention are controlled by cell adhesion molecules, i.e., selections, integrins and members of the immunoglobulin superfamily (Barthel et al., 2008; Barnes, 2011). The expression and function of these adhesion molecules and the subsequent chemotactic attraction and activation of infiltrating pro-inflammatory cells are controlled by a myriad of cytokines, chemokines and mediators with the Th2 cytokines IL-4, IL-5, and IL-13 representing essential and central coordinators of asthmatic inflammation (Barrett and Austen, 2009; Walsh, 2010). Thus, modulating the cytokine network in asthma with biological therapy targeted to patients with particular eosinophilic phenotypes represents a plausible paradigm for treatment of this important condition (Petsky et al., 2007; Barnes, 2008; Desai and Brightling, 2009; Walsh, 2011).

## Interleukin-5

The crucial role of IL-5 in the development and release of eosinophils from the bone marrow, their enhanced adhesion to endothelial cells lining the post-capillary venules and their activation and secretion in the tissues has been known for many years (Egan et al., 1996). IL-5 was identified as a target to prevent or blunt eosinophil-mediated inflammation in patients with asthma, leading to the development of humanized anti-IL-5 mAb such as mepolizumab, reslizumab and benralizumab (Long, 2009; Molfino et al., 2012). Early clinical trials with mepolizumab (GlaxoSmithKline plc) reported significant reductions of blood and sputum eosinophils but clinical outcomes were disappointing (Leckie et al., 2000; Flood-Page et al., 2003; Kips et al., 2003; Flood-Page et al., 2007). However, these studies all used subjects recruited on the basis of clinical and physiological characteristics not associated with the presence of eosinophilic airway inflammation (O'Byrne, 2007). Two more recent studies in highly selected asthma patient populations with a demonstrable sputum eosinophila reported that mepolizumab treatment not only reduced eosinophil numbers in the blood and sputum but also gave a significant reduction in asthma exacerbations and also demonstrated that mepolizumab attenuates aspects of eosinophil-induced airway inflammation refractive to glucocorticoid (GC) therapy (Haldar et al., 2009; Nair et al., 2009). A recent randomized, placebo-controlled trial evaluated intravenous reslizumab in patients with poorly controlled asthma, a sputum eosinophilia greater than 3% and who were taking high-dose inhaled GC. The reslizumab group exhibited a significant decrease in sputum eosinophilia with a non-significant trend toward improvement in asthma control with a significant improvement in lung function as assessed by the Asthma Control Questionnaire score. In patients with concomitant nasal polyposis, reslizumab treatment was associated with a significant improvement in asthma symptoms. There was a non-significant reduction in asthma exacerbations in the reslizumab group while the adverse-event profile for reslizumab and placebo were similar (Castro et al., 2011). Benralizumab is a novel humanized afucosylated IgG1κ mAb indicated for the potential treatment of asthma and COPD that binds to a distinct epitope within the extracellular domain of recombinant human IL-5Rα (Ghazi et al., 2012). Afucosylation is associated with enhanced antibody-dependent cell cytotoxicity and benralizumab was found to potently induce apoptosis in eosinophils and basophils (Kolbeck et al., 2010). Tissue eosinophils in bronchial biopsies of patients with mild atopic asthma exhibited intense immune positivity for benralizumab in contrast to resident mast cells, which were negative. Other anti-IL-5 mAb act by neutralizing the effects of IL-5. In contrast, benralizumab targets effector cells, mainly eosinophils, and basophils. A phase-1 study in subjects with mild asthma demonstrated that intravenous benralizumab rapidly induced near total depletion of peripheral blood eosinophils while exhibiting an adequate safety profile and dose-proportional pharmacokinetics (Busse et al., 2010).

## Interleukin-4 and -13

Both IL-4 and IL-13 are important in eosinophil accumulation and are key factors in IgE synthesis by B cells (Wills-Karp et al., 1998) together with direct effects on airway epithelial cells (Kuperman et al., 2002). Several clinical trials of anti-IL-13 biologics reported disappointing clinical outcomes although this cytokine is widely regarded as central to asthmatic inflammation. However, the latter conclusion is almost exclusively based on studies that used animal-models of asthma. Indeed, out of around 3000 peer-reviewed publications implicating IL-13 as a central mediator in asthma, only four relate to direct evidence in human asthma (Holgate, 2010). Importantly, in two of these studies, elevated levels of IL-13 were present in the sputum of around half of asthmatic patients tested with no significant association with disease severity (Berry et al., 2004; Saha et al., 2008). It is not surprising therefore that anti-IL-13 proved to be disappointing in asthma therapy given that only half of the patients recruited to a given clinical trial have any likelihood of responding to this biologic. The anti-IL-13 mAb lebrikizumab (Genentech/Chugai Pharmaceutical) has recently been demonstrated to significantly improve lung function in patients with inadequately controlled asthma, but only in a subgroup defined on the basis of high serum levels of periostin (Corren et al., 2011). The latter is a cellular matrix protein that is released by airway epithelial cells stimulated with IL-13. Periostin exhibits effects on epithelial cells and fibroblasts that may contribute to airway remodeling in asthma (Takayama et al., 2006; Sidhu et al., 2010) and may prove to be useful as a biomarker to identify those subjects most likely to give a positive clinical response to IL-13 antagonism. The recombinant human IL-4 variant, pitrakinra (Aerovant) competitively inhibits the IL-4Ra receptor complex to interfere with the actions of both IL-4 and IL-13 and initial clinical trials indicated that such an approach may prove beneficial in patients with atopic asthma (Walsh, 2012). For example, a placebo-controlled trial of inhaled pitrakinra in 534 patients with uncontrolled, moderate-to-severe asthma reported significant effects on exacerbations rates and symptom scores in patients with an elevated blood eosinophilia (Wenzel et al., 2010). In contrast, a study that examined AMG 317, a fully humanized mAb to IL-4Rα, in patients with moderate to severe asthma reported no significant effects on primary or secondary clinical endpoints. However, clinically significant effects were observed in those patients who had the most uncontrolled or symptomatic asthma (Corren et al., 2010). It is interesting to note that analysis of single nucleotide polymorphisms leading to for amino acid changes in the 3′ end of the IL-4R α gene identified a sub-group of patients with moderate-to-severe asthma who were significantly more responsive to pitrakinra therapy (Slager et al., 2012).

## Tumour necrosis factor-α

Tumour necrosis factor (TNF)-α, an important cytokine in innate immune responses, has been implicated in several chronic inflammatory diseases including rheumatoid arthritis and Crohn's disease with anti-TNFα therapy proving useful in these conditions. It is produced principally by macrophages while other pro-inflammatory cells including monocytes, dendritic cells, B lymphocytes, T cells, neutrophils, mast cells and eosinophils, together with the structural cells fibroblasts, epithelial cells, and smooth muscle cells represent significant sources. TNF-α has pro-inflammatory effects on eosinophils, neutrophils, T cells, epithelial cells and endothelial cells and may play a key role in amplifying airway inflammation through activation of transcription factors such as NF-κB and AP-1. TNF-α is expressed in biopsies and lavage fluid from asthmatic airways, particularly in patients with severe asthma compared with those with well-controlled disease. TNF-α is thought to contribute to AHR, airway remodeling and GC resistance in asthma and therefore represents a potential target for therapy (Walsh, 2011). Humanized murine anti-TNF mAb (infliximab) and soluble TNF receptor linked to human IgG1 (etanercept) have been developed and preliminary clinical studies in asthma did show significant improvements in lung function, airway hypereactivity and exacerbation rate, particularly in patients with severe asthma refractory to GC treatment (Brightling et al., 2008). However, a more recent clinical trial in 132 subjects with moderate-to-severe persistent asthma reported no significant differences between etanercept and placebo for any efficacy end-points although etanercept was well-tolerated. In addition, the anti-TNF biologic golimumab in patients with severe uncontrolled persistent asthma also reported negative clinical findings. Importantly, this study was terminated early due to unacceptable adverse events including frequent serious infections, eight malignancies and one death in the active-treatment group compared with the placebo group (Wenzel et al., 2009). Overall it appears that TNF-α inhibitors are effective in a sub-group of patients with asthma, and again identification of the correct patient population may improve clinical outcomes (Matera et al., 2010). However, the unfavorable risk/benefit ratio exhibited by golimumab does cast doubt on the future of anti-TNF therapy in severe asthma.

## Conclusion

The development of novel asthma anti-inflammatory therapy based on targeting cytokines for the most part initially proved to be disappointing. The majority of biologics have proven inadequate in the clinical setting in asthma even though they were highly effective in animal models of asthma, most likely because the artificially-induced airway inflammation in the latter is not a true representation of the wide spectrum of pathology observed in asthmatic patients. The development of discriminatory biomarkers and genetic profiling may identify patients with particular sub-phenotypes of asthma allowing therapy to be targeted to those most likely to exhibit beneficial effects. This combined with the development of biologics aimed at the antagonism of more than one cytokine, as exemplified by pitrakinra, may prove a more effective approach.
